# Selections and Abstracts

**Published:** 1898-02

**Authors:** 


### SELECTIONS AND ABSTRACTS.

### IRREGULAR MENSTRUATION IN YOUNG WOMEN DUE TO ANEMIC CONDITIONS.

The young physician just starting into practice cannot help but be impressed with the frequent occurrence of menstrual disorders in young girls during the period just succeeding the age of puberty. The metamorphosis of a girl into a woman, consisting as it does of structural and functional changes throughout her body, in many instances leaves behind pronounced alterations in the quality or even quantity of the blood current. How common it is to have a mother bring her daughter to the physician and say, "Doctor, I would like to have you do something for my daughter. For nearly a year she has been losing interest in everything, and seems to be completely worn out. She has no appetite and absolutely no ambition for work, study or play. She does not lose flesh or grow thin at all, but her color is so poor and she seems so weak that I fear she is going into consumption."

Inquiry on the part of the doctor elicits the further information that the young lady in question is sixteen years old or thereabouts, and that she is a schoolgirl. A year or two ago she first menstruated and since that time has been unwell only twice, or at irregular intervals varying anywhere from three to nine months. Her bowels are either constipated, or the reverse, and she may complain of headaches, vertigo, palpitation of the heart, insomnia, indigestion, etc. The pale face with its sallow greenish tinge, the bleached tongue, the colorless conjunctivæ and finger nails, tell well the tale of impoverished blood. Combine the history with the objective symptoms and the diagnosis is clear of chlorosis or green-sickness. The absence of cough or pulmonary symptoms excludes the dreaded "consumption," but we have instead a condition of the blood in which the essential constituents are diminished and the whole quality of the life-giving current so depreciated that the various organs of the body are unable to perform their normal functions. The uterus is small and illy developed, and the supply of rich blood it so urgently requires in its developmental state is not to be had. Is it any wonder, then, that the chlorotic girl does not menstruate regularly? It is a great wonder that she ever menstruates at all. Correct the anemic or impoverished condition of her blood and the physiological function of her uterus will be resumed as naturally as that of any other organ.

How this chlorotic condition can best be corrected is the next question, and one which because of its frequency concerns every practicing physician. Countless remedies have been presented to the profession, but far and foremost above them all is iron, notwithstanding certain high authority to the contrary. Arsenic is certainly valuable, but it ranks far below iron or even manganese in therapeutics of anemia. In order to be most efficacious, however, the iron should be in its most readily assimilable form, and until recently the carbonate and albuminate have been supposed to present this requisite in the highest degree. But since manganese has grown in favor as an adjuvant to iron, a new preparation has been submitted to the medical profession, *and in every way it has proven itself an ideal one.* I refer to Dr. Gude's preparation of the peptonate of iron and manganese, known as Pepto-Mangan. This admirable combination of iron and manganese is readily taken into the human economy and appropriated to its needs, without deranging the weakest alimentary tract, or hindering in any way the normal processes of digestion, assimilation and excretion. It should be given in water or milk in teaspoonful doses after meals, and its administration is invariably followed by the results desired.

But in order that the medical treatment of chlorosis may be most valuable and efficient, it should be augmented by auxiliary treatment consisting of careful attention to diet and exercise. It goes without saying that the food of an anemic girl should be most nutritious and particularly abundant in albumen, while the exercise should aim to provide greater quantities of oxygen in the form of pure air, without lowering the vitality. Walking, skating, tennis or bicycling in moderation are all able to supply the demand for exercise.

Treatment laid down on the above lines, followed out in every instance with good habits of hygiene and a careful observance of Nature's demands, will regulate the various functions of the body, and the menstrual function will prove no exception to the rule. —*H. Edwin Lewis, M.D., Vermont Medical Monthly, August, 1897.*

---

### THE VALUE OF POTASSIUM PERMANGANATE AS AN ANTIDOTE FOR MORPHINE.

In the last *Therapeutic Gazette* Drs. Thornton and Holder, of Philadelphia, publish the results of some experiments upon dogs, which were practiced to ascertain the true value of permanganate of potash as an antidote for opium and morphine.

The power of potassium permanganate to oxidize morphine and some other alkaloids, when brought in direct contact with them, is not to be disputed, and if administered by the mouth in dilute solution while opium or its alkaloids are still in the stomach, immediately oxidizes them and thereby prevents toxic effects from being manifested. The originator of the antidote as well as several other physicians appear not to be satisfied with the beautiful and satisfactory action of the antidote in neutralizing the effects of morphine when administered by the mouth, but put forward the extravagant claim that even when given hypodermically potassium permanganate was a valuable antidote to opium and its alkaloids.

For the drug to be of value when given hypodermically there are but three possible ways in which it may overcome the poisonous effects of opium: First, chemically, by coming in contact with the alkaloids in the blood and thereby oxidizing them; second, by acting as a physiological antagonist; and, third, mechanically, by giving rise to severe pain which follows the injection of potassium permanganate, thereby assisting in keeping the patient awake.

To substantiate or disprove these claims these experiments were undertaken. The toxic dose of morphine sulphate for dogs was found to be about .750 gram per kilogram of body weight. This dose was usually fatal in from two to three hours. Then five dogs were given toxic (hypodermic) doses in the same manner, and followed by subcutaneous injections of potassium permanganate in from five minutes to an hour, and still all the animals died as promptly as if no permanganate had been given.

Next, a dog was given less than a toxic dose of morphine hypodermically and the antidote given. The effects of the morphine were the same as if no potassium permanganate had been administered.

Finally, a dog to which no morphine had been administered was given twenty grams of potassium permanganate subcutaneously, and the only effect noted was that the injection gave rise to symptoms of very severe pain, which passed off in about thirty minutes.

One grain of potassium permanganate is said to neutralize one grain of morphine, but in test tubes at least two grains of potassium permanganate are required to reduce one grain of morphine, and these experiments were conducted upon this basis.

The authors then give in detail their observations upon these dogs, and conclude by saying that "these experiments lead to most serious doubt as to the efficiency of potassium permanganate as an antidote to opium or its alkaloids when given hypodermically."

---

### POST-MORTEM DELIVERY.

An account of one of the rare cases of spontaneous delivery of a child some days after the death of the mother appeared in the last number of the *Viertelgahrschrift fur Gerichttiche Medicin*, and has given rise to considerable comment. The case is very well authenticated, as the body passed through the hands of medical and legal public officials before burial, so that the details are a matter of legal evidence.

The woman had been found dead in the river under circumstances which seemed to point to suicide. Careful examination of the body was made to be sure that there was no evidence of injury previous to the immersion, and it was noted that she was in an advanced stage of pregnancy. Ten days later circumstances came to light which seemed to point to foul play, and the body was exhumed for further and detailed examination.

A tumor was found projecting from the vulva which had not existed at the time when she was placed in the coffin. This was found to consist of the completely inverted uterus and vagina. A little below it, lying upon the lower limbs, was a fetus 36 cm. in length.

Dr. Bleich, who reported the case, discussed the possible causes of the delivery, and considered that the usual causes do not afford a sufficient explanation. Neither the development of gas within the abdomen and the consequent pressure exerted upon the fundus uteri nor tonic contraction of the uterus due to *post-mortem* rigidity are sufficient explanation of this remarkable phenomenon; as far as he is concerned, neither nor both of them can account for it, if one is to consider the uterus as being in its ordinary *post-mortem* condition. He considered it probable that during the death agony there had been active contractions of the uterus which had almost accomplished the extrusion of the child, and then that the pressure of putrefactive gases in the abdomen and the further tonic contraction of the uterus were sufficient to have completed the process of delivery.—*Berlin Correspondent of Medical News.*

---

### A NATIONAL QUARANTINE LAW.

Senator Caffery, of Louisiana, has passed through a disagreeable experience this summer, which may prove of great benefit to all the Gulf States. After suffering a great deal of personal inconvenience and vexation by reason of the local quarantine regulations, he came to a realizing sense of the vast importance of a proper national quarantine. As a result, he has introduced into the United States Senate a well-drawn and vigorous measure providing for the extension of the quarantine powers of the Marine Hospital Service.

The bill in question makes the National Quarantine as administered by the Marine Hospital Service paramount to all local quarantine boards, so that no ship can enter any port without submitting to the regulations of the Treasury Department, as administered by the Marine Hospital Service; nor can any ship be detained for quarantine inspection after it has been passed by the Marine Hospital authorities. The bill also provides that whenever yellow fever, cholera, plague, or typhus fever has passed the quarantine of the United States, or in any manner any one of these diseases has gained entrance or has appeared within the limits of any State or Territory, the quarantine regulations of the United States, prepared under the direction of the Secretary of the Treasury, shall be supreme and have precedence of State or municipal laws, rules, or regulations. The President is authorized to enforce the same within the limits of any State or Territory, to control the government of vessels, railroad trains, vehicles, or persons within any State or Territory, to prevent these diseases from spreading from one State or Territory to another State or Territory, and to prevent unnecessary restrictions upon interstate commerce. —*Gaillard's Medical Journal.*

---

### THE NATIONAL QUARANTINE BILL.

Senator Caffery, of Louisiana, has introduced a bill in the United States Senate which will provide for national control of quarantine regulations. The bill makes the national quarantine as administered by the Marine Hospital Service paramount to all local quarantine boards, so that no ship can enter any port without submitting to the regulations of the Treasury Department, as administered by the Marine Hospital Service, nor can any ship be detained for quarantine inspection after it has been passed by the Marine Hospital authorities. The bill also provides that whenever yellow fever, cholera, plague, or typhus fever has passed the quarantine of the United States, or in any manner any one of these diseases has gained entrance or has appeared within the limits of any State or Territory, the quarantine regulations of the United States, prepared under the direction of the Secretary of the Treasury, shall be supreme and have precedence of State or municipal laws, rules, or regulations. The President is authorized to enforce the same within the limits of any State or Territory, to control the government of vessels, railroad trains, vehicles, or persons within any State or Territory, to prevent these diseases from spreading from one State or Territory to another State or Territory, and to prevent unnecessary restrictions upon interstate commerce.

The people of the States recently afflicted by the scourge of yellow fever cannot shake off the horrors and hardships of that calamity, and look forward with dread and apprehension to the return of warm weather. The Marine Hospital Service is already organized on a sufficient comprehensive basis to enable it, promptly upon the enactment of this bill, to extend its equipment and be ready at the first indication of an awakening of the dread disease to fulfil efficiently all the requirements of its new functions. By the passage of this bill without undue delay, the existing confidence in the uniform vigorous enforcement of quarantine and of sanitary regulations which has characterized the work of the Marine Hospital Service, will restore a feeling of security to the business interests of the South and of safety to the people.—*Medical News.*

---

### FOR REMOVAL OF WARTS AND CORNS.

℞. Acid salicylic.......................................ʒ ss.

Ext. cannabis indic.......................................gr. v.

Collodion flex.......................................℥ ss.

Sig.: To be painted over such an area, twice daily, for three days, when an oil poultice is applied over night.

—*Med. Review of Reviews.*

---

### WHEN SHALL WE USE THE FORCEPS.

Dr. Park (*Am. Gyn. and Obstet. Journ.*) lays down the following principles: 1. Indications for the use of forceps rarely arise in the first stage of labor before the membranes have been ruptured. 2. It may be necessary to employ the forceps during the first stage, when the waters have escaped on account of the increasing exhaustion of mother and child. 3. It is proper during the first stage of labor to apply the forceps for accidents, whenever they may arise, notably in certain cases of convulsions, placenta previa and prolapse of the cord. 4. In the second stage it is proper to apply the forceps half an hour after the head ceases to advance, when there is no disproportion between the passage and the passenger. 5. When, however, there is a tight fit between the child and the birth canal, the use of the forceps may be delayed. This delay should rarely exceed two hours after the head ceases to advance. 6. If the head is engaged, and neither advances with the pain nor recedes after the pain, the forceps should be promptly applied.—*Med. Standard.*

---

### REQUIREMENTS OF THE VARIOUS STATES REGARDING THE LICENSE TO PRACTICE MEDICINE.

To obtain a license to practice medicine it is necessary to pass an examination in the following States: Alabama, Delaware, District of Columbia, Florida, Georgia, Idaho, Louisiana, Maine, Maryland, Massachusetts, Minnesota, Mississippi, Montana, New Hampshire, New Jersey, New York, North Carolina, North Dakota, Oregon, Pennsylvania, South Carolina, Utah, Virginia, Washington, and West Virginia. Examination is required although certain diplomas are recognized as an equivalent in the following States: Arkansas, Colorado, Connecticut, Illinois, Indiana, Iowa, Missouri, Oklahoma, Rhode Island, Tennessee, Vermont, Wisconsin, New Mexico. A diploma only is required in the following States: California, Kentucky, Nebraska, Ohio, South Dakota, Texas (practically). The law practically imposes no restriction in the following: Alaska, Arizona, Kansas, Michigan, Nevada, Wyoming. Dr. D. C. Newman of Seattle, Wash., writes us that the law requiring a license to practice medicine in that State is practically a dead letter, for it is never enforced, and adds that fully one-fourth of the practising physicians of that State have never complied with it.—*Med. News.*

---

### BABY INCUBATION IN NEW YORK.

There has been established in New York a Lion's Infant Incubator Institute, an American branch of the *Maternité Lion*, of Paris, which will undertake to rear premature children under ordinary circumstances of nursing non-viable. Professor Lion first demonstrated the value of his incubator in Marseilles some years ago, and institutes were established there and at Nice and Lyons. The fame of Professor Lion's apparatus now reached Paris, and leading physicians at the French capital induced the inventor to establish an infant-saving institution in that city. His success here was such that the municipal government, in recognition of the boon he had conferred upon humanity, unanimously voted an annual subsidy for the maintenance of his establishment, as well as for the employment of his incubators in all of the maternity hospitals of that city. The Lion Institute in Paris has reduced the annual death-rate of "prematures" from eighty per cent. of two years ago to seventeen per cent. last year. It was here that American physicians touring Europe for pleasure visited the Lion Institute, and urged the Professor to establish a branch of that institution in New York. Professor Lion is now in New York directing an institute which for beauty, convenience, and effectiveness has no superior elsewhere, and it has already a half-dozen living mites, averaging two pounds each in weight.

---

### SUPRAPUBIC CYSTOTOMY.

Professor John Wyeth, in the *New York Polyilinic*, in speaking of supra-pubic cystotomy, says:

1. The parts about the field of operation and where the urine might flow, should be shaved.
2. The patient should rest in full extension upon a table.
3. It is of advantage to have the hips higher than the shoulders, as the weight of the intestines will be thus removed from the bladder.
4. Rectal distension is never necessary.
5. Water is the best thing with which to distend the bladder.
6. The longitudinal incision beginning one inch above the margin of the symphysis pubis and two inches long, is the best.
7. Arrest hemorrhage as the operation proceeds.
8. The size of the incision in the bladder must be regulated to suit the individual case.
9. The peritoneum should be dissected up from the bladder and if cut or torn should be sutured at once with catgut.
10. Close the opening in the bladder when the case will admit of it by using catgut and the Lembert suture.

11. Pack the superficial opening with iodoform gauze and let it close by granulation.

12. The bladder must be kept empty for three days, either by frequent catheterization or by leaving the instrument tied in the bladder.—*Medical and Surgical Bulletin.*

---

### HYSTERIA DUE TO PELVIC DISEASE.

It is very common in women suffering from pelvic disease to find more or less pronounced symptoms of mental depression. They are brooding, despondent and frequently given to tears. For the relief of this condition, along with change of environment and directions for plenty of outdoor exercise, Dr. Talley found the following formula of service:

| | | |
|---|---|---|
| ℞ | Strychnin sulph. | gr. 1-42. |
| | Quinin sulph. | gr. iss. |
| | Ext. hyoscyam. | gr. iss. |
| | Ferri redact | gr. i. |

M. For one pill. Dose.—One pill thrice daily.—*Polyclinic.*

---

### IS CRIME A DISEASE?

Of 394 thieves, 74 are dolichocephalic, 129 mesocephalic, 191 brachycephalic; of 107 homicides, 21 are dolichocephalic, 31 mesocephalic, 54 brachycephalic; of 92 sexual offenders, there are 18 dolichocephalic, 30 mesocephalic, 38 brachycephalic; of 54 swindlers, there are 9 dolichocephalic, 15 mesocephalic, 30 brachycephalic. A study of the individual indices shows a considerable proportion to be entirely outside of the physiologic limit. This is most marked among the sexual offenders, in whom the cephalic index was in itself absolutely pathologic in about 15 per cent.; that is to say, considering the anteroposterior diameter of the cranium as 100, then the transverse was represented by less than 76 or more than 87, the former being extremes of dolichocephalic and the latter of brachycephalic skulls. The brachycephalic skulls much predominated in the entire group of criminals.—*Medical Record.* This proves what has been known for ages, namely that the long-headed individual is the best equipped for the struggle for existence.—*Cleveland Journal of Med. and Surg.*

### ZINC PERMANGANATE IN GONORRHEA.

A. S. Hosaling, in the *Med. News* (LXIX.), states that the remedy that has given him the most satisfaction in the largest number of cases of gonorrhea, besides being equally successful in both the acute and chronic forms, is zinc permanganate. One remarkable characteristic of its action is the almost total absence of irritation following its use, the patient seldom complaining of pain. Its effects are discernible almost immediately, the discharge in the majority of cases becoming greatly reduced after a few injections.

The method of treatment is simple:

After the stage of acute inflammation has subsided, the injections are made four and five times a day after urination, with an ordinary blunt-pointed hard-rubber syringe, having a capacity of from three to four dr.

Treatment is begun with a solution of ½ grn. to 1 fl. oz. of water, gradually increasing it to 1½ grn. An alkaline diuretic is given, and the hygienic part of the treatment, which is of the utmost importance, is followed closely in every case.—*Am. Med. Surg. Bulletin.*

---

### INDICATIONS FOR THE INDUCTION OF ABORTION.

Dr. Jeffé (*Medicinische Neuigkeiten*), after a study of the literature of the last ten years, fixes the indications for inducing abortion as follows: (1) Uncontrollable vomiting of pregnancy. (2) Incarceration of the gravid uterus. (3) Obstruction of the pelvic outlet by tumors or exudates. (4) Progressive and pernicious anemia. (5) Grave chorea. Relative indications: (1) Great contraction of the pelvis with the conjugata vera below five centimeters. (2) Pulmonary emphysema, with signs of degeneration of the heart. (3) Nephritis, especially with eclampsia. (4) Chronic heart disease. (5) Other general diseases of the mother which would jeopardize her life at the time of delivery. The author holds that a conjugata vera of six centimeters and advanced pulmonary tuberculosis should not be regarded as indications for abortion, as it is not just to sacrifice a future life for one that is "certainly lost."—*Med. Times.*

THE Christian Scientists have received what to them is a rather serious set-back in the State of Pennsylvania. They wanted a charter for the First Church of Christ Scientist, which the court refused to grant. The judge distinctly declared that if they were a purely religious and teaching body the Constitution would guarantee perfectly their liberties in such directions, but when they wanted a charter to give them the privilege to violate the laws of the State, no such charter could be granted. He showed that for them to treat small-pox, consumption, cancer, or scrofula would be a violation of the act of March 24, 1877, that demands a proper education of every person who undertakes to treat disease.—*Medico-Surgical Bulletin.*

---

IN further effort to prevent the invasion of the United States by yellow fever the importance of the discovery of the exact cause of the disease, which up to the present time has been undetermined, is obvious, and to this end a systematic bacteriological investigation should be made. I therefore recommend that Congress authorize the appointment of a commission by the President, to consist of four expert bacteriologists, one to be selected from the medical officers of the Marine Hospital service, one to be appointed from civil life, one to be detailed from the medical officers of the army, and one from the medical officers of the navy. —*From the President's Message.*

---

WHEN you wish to pack a large cavity with gauze, a good way is to fit a single layer or sheet of the material all round against the walls of the space, allowing the edge of the gauze to remain outside. You then have a gauze bag lining the cavity. Into this bag you may pack as much gauze as may be necessary for pressure, or for thorough absorption of the discharges. When the time comes for removing the packings you will find that by first removing the inner filling of the gauze bag, and last of all the bag itself, there will be comparatively little pain, little hemorrhage, and little derangement of the tissues.—*Internat. Jour. of Surgery.*

### AGREEABLE SALICYLIC MIXTURE.

℞ Potassii acetatis..................................℥ ij.
Acidi salicylatis..................................℥ ss.
Syr. limonis..................................℥ ij.
Aq. menth. pip..................................℥ viij.

Sig. Tablespoonful every three hours.

—*North Amer. Prac.*

---

HEADACHES, if due to pelvic disturbances in the female, are usually located at the top of the head and are accompanied by soreness of the scalp; if due to digestive disturbances, they are occipital or frontal; if to disease of the pharynx, they involve the entire vault as though the pharynx were expanded and extended upward; if due to migraine, they are usually one-sided, local, and accompanied by soreness at the superaorbital foraman: if to eye-strain, generally superciliary or frontal, sometimes occipital; if to disease of the nares, between the eyes and extending backwards. Dercum. —*Medical Record.*

---

DO WE need a new and revised code of ethics to meet these constantly multiplying exigencies? Probably so. The times are now different from those of the birth of the old code. However, I am inclined to accept the version of that great and good old man, Dr. D. W. Yandell, who used to say to his classes of medical students, when speaking of the code, "Why, gentlemen, what need have we for a code of ethics? A gentleman never needs a code of ethics to govern his actions, and a d—— rascal will never live up to a code."—*The Am. Therapist.*

---

AN Arkansas hotel was advertised as follows: "This hotel will be kept by the widow of the former landlord, Mr. Brown, who died last summer on a new and improved plan."—*Milwaukee News.*

